# New Implant Materials

**DOI:** 10.3390/ma16134525

**Published:** 2023-06-22

**Authors:** Filiberto Mastrangelo

**Affiliations:** Clinical and Experimental Medicine Department, University of Foggia, via L. Rovelli n.48, 71122 Foggia, Italy; filiberto.mastrangelo@unifg.it

In the last forty years, dental implantology has become a widespread worldwide clinical practice in medicine, able to rehabilitate partial or full human edentulism of the jaw and highly successful over the long term [1]. However, several present and future challenges for clinicians remain extant, related to the minimal invasivity of the surgical procedures, the long-term maintenance of the results, the predictability of the results in patients with co-morbidities and in conditions with hard and soft deficiencies, the time reduction in clinical rehabilitation, the reduction in the cost of biomaterials, the sustainability of the production methods of biomaterials and the successful esthetic results in relation to to hard and soft tissue in particular in the esthetic areas of the smile. The success of dental implants relies on the biological process of osseo-integration and recently soft-integration, which is a crucial factor in the long-term stability of dental implants [2,3]. This process can take several months, which can be a major inconvenience for patients. To meet and overcome these challenges, several elements of research and innovative surgical procedures have been promoted and various devices, materials and bio-materials have been produced to achieve minimally invasive, predictable, immediate or long-term results and to understand the biological properties and characteristics, in physiological and pathological conditions, able to play a crucial role in bone and soft tissue healing, regeneration and human body bio-materials’ integration [4,5]. For a long time, implant macro- and micro-design structures were considered as the focus of the efforts of the researchers trying to study the best characteristics to obtain high clinical rates for dental implant rehabilitations. Once predictable results were obtained, the focus of the research was aimed at understanding how to reduce the osseointegration times of the fixture [6]. In this phase, several studies were developed, which have led to new micro-rough surfaces capable of improving the adhesion of the bone to the implant defined by the Bone to Implant Contact percentage (BIC) with a relevant time reduction in osseointegration [7]. After these results, several researchers have developed clinical studies able to promote early-loading implant rehabilitations related to innovative technological developments both of the intra-osseous macroscopic component of the implants and of the extra-osseous macroscopic component in connection with the prosthesis [8]. However, both qualitative and quantitative bone loss, in several areas of the jaw, especially in patients who have had tooth loss for a long time, have promoted a series of in vitro and in vivo studies to regenerate bone tissue capable of developing new surgical techniques for three-dimensional bone volumes reconstruction in the different areas of the atrophic maxilla to obtain, with a “prosthetic guided surgery”, an adequate dental implant osseointegration [9]. In this period, several studies were conducted to understand which were the best techniques to obtain predictable results through the use of autologous grafts from intra- or extra-oral donor sites and which were the ideal characteristics for the best autologous graft [10]. The requirement to perform invasive operations on donor sites for the reconstruction of atrophic jaw areas has reduced these bone surgical techniques to use only in the presence of severe jaw atrophies, allowing for the development of research and technologies capable of bone repair and reconstruction through reabsorbable or non-reabsorbable autologous or/and heterologous bio-materials [11]. In the same period, another line of research was aimed at developing the tissue engineering field, which through the use of several innovative biomaterials, growth factors and stem cells at the same time, discovered it was possible to promote a real hard-tissue regeneration, avoiding surgery of donor sites and intra- and post-operative complications [12]. During the 1990s, the patients’ requests to obtain an immediate loading implant rehabilitation promoted several studies in developing innovative surfaces- micro- and nano-roughness characteristics through the use of new technologies for the production of implant surfaces. Recently, several studies have evaluated the bone behavior, the cell ultrastructure aspects, and the biological tissue response in relation to the different surface micro- and nano-roughness characteristics [13,14]. In addition, surface nanostructures seem to increase the surface energy and wettability and seem able to mime the extracellular matrix capability to increase the interactions first with integrins and then with cells during the first phase of bone formation and implant osseointegration [15,16,17]. This new point of view is of relevant interest in improving healing patterns around implants and trying to speed up the healing processes [18,19]. Now,

A further aspect of great interest is the controlled release of anti-inflammatory factors. This would allow diffusion over time in a controlled way, through the use of resorbable scaffolds. This drug-delivery system can improve the capabilities of biomaterials and implants where there is an important inflammatory or infectious phenomenon with a reduction in post-surgical complications and morbidity [20]. In the last ten years, the requirement to reduce the costs of rehabilitation and reduce the number and the morbidity of surgical interventions (minimal invasiveness) allowing a greater number of patients to obtain a fixed prosthesis on implants, has developed surgical procedures with a reduced number of immediately loaded implants (all-on-four and all-on-six) and post-extraction interventions, without the need to use biomaterials. Further developments of minimally invasive procedures have been promoted with the use of digital dentistry software which has made it possible to design and create guided implants inserted with flapless surgeries. The growing diffusion of some religious and animalist cultures has also shifted the attention of research towards biomaterials not derived from animals. The use of synthetic or autogenous-derived biomaterials seems to be an important trend in research [21]. The tooth as grafting material seems to provide excellent osteoconductivity capabilities to which is added the osteoinductive role exercised by the BMP-2, Col-1, VEGF, PDGF, etc., present in it [22]. A possible field of interest is also the combination of platelet concentrates and stem cells to dental implants [23,24] to obtain an improvement in long-term results and a reduction in osseointegration time. In the future, there will certainly be further developments of products, devices, and biomaterials able to promote the sustainability of production methods as well as the scientific community providing innovative products and procedures for esthetic success in the integration of hard and soft tissue implants, especially in esthetic areas. In the next few years, it will be necessary to increase the in vivo studies to understand the behavior of different biomaterials in relation to the bone and the relationship between these and the implant screw. Another relevant aspect will be the study of the human body’s immunological and biological response to the titanium and the different biomaterials. Several studies in humans will be necessary to understand the dental implantology predictability not only in healthy patients but also the long-term success rates in patients with co-morbidities such as diabetes, osteoporosis, neoplastic pathologies, allergies, nephropathies, heart diseases and if and how single or multiple drug therapies could influence the osseointegration, the bone remodeling and the development of mucositis and peri-implantitis. New metal alloys and bio-ceramics, innovative devices, polymers and nanoparticles, hybrid composites, novel macro- and micro-design materials, new drugs, innovative equipment for diagnosis and clinical applications, and innovative chemicals or physical substances that promote or improve bone and soft tissue response are among the topics that are being researched to achieve patient satisfaction.

From this perspective, the Special Issue of Materials will collect original high-quality papers of the most recent and advanced research, including experimental research results in vitro and in vivo on animal and human models, as well as literature reviews and meta-analysis. This collection aims to provide crucial improvements in the knowledge of novel biomaterials, drugs, and technology for implantology and medicine.

To achieve this goal, all medical specializations are invited to contribute to this collection. The collection aims to provide a platform to showcase the most recent and innovative research in the field of dental implantology. The innovative research strategies in the biomaterials field and innovative biomedical technology are today focused on reducing the time of clinical rehabilitation, providing immediate or long-term activity, and controlled properties capable of playing a critical role in human bone and soft tissue healing and regeneration.

One of the key focuses of this research is to develop minimally invasive procedures to increase patient compliance. The ability to reduce the time of clinical rehabilitation and minimize the discomfort of patients will be a major advancement in the field of dental implantology. Additionally, the development of innovative materials and bio-materials will provide more efficient and effective ways of promoting and improving bone and soft tissue response in physiological and pathological conditions towards implants or prostheses.

In conclusion, this Special Issue of Materials will be a valuable collection of research that will provide crucial improvements in the knowledge of novel biomaterials, drugs, and technology for implantology and medicine. This collection will showcase the most recent and innovative research in the field of dental implantology and will help to reduce the time of clinical rehabilitation, increase patient compliance, and provide more efficient and effective ways of promoting and improving bone and soft tissue response.
